# A Tumor-Specific Neo-Antigen Caused by a Frameshift Mutation in *BAP1* Is a Potential Personalized Biomarker in Malignant Peritoneal Mesothelioma

**DOI:** 10.3390/ijms17050739

**Published:** 2016-05-14

**Authors:** Jun Lai, Zhan Zhou, Xiao-Jing Tang, Zhi-Bin Gao, Jie Zhou, Shu-Qing Chen

**Affiliations:** 1 Institute of Pharmaceutical Analysis and Drug Metabolism, College of Pharmaceutical Sciences, Zhejiang University, 866 Yuhangtang Road, Hangzhou 310058, China; laijun@zju.edu.cn (J.L.); zhouzhan@zju.edu.cn (Z.Z.); txjgyqy@163.com (X.-J.T.); 2 Department of Obstetrics and Gynecology, Yuyao People’s Hospital, 800 Chengdong Road, Yuyao 315400, China; gzb-gx@163.com

**Keywords:** neo-antigen, BAP1, personalized immunotherapy target, mesothelioma

## Abstract

Malignant peritoneal mesothelioma (MPM) is an aggressive rare malignancy associated with asbestos exposure. A better understanding of the molecular pathogenesis of MPM will help develop a targeted therapy strategy. Oncogene targeted depth sequencing was performed on a tumor sample and paired peripheral blood DNA from a patient with malignant mesothelioma of the peritoneum. Four somatic base-substitutions in *NOTCH2*, *NSD1*, *PDE4DIP*, and *ATP10B* and 1 insert frameshift mutation in *BAP1* were validated by the Sanger method at the transcriptional level. A 13-amino acids neo-peptide of the truncated Bap1 protein, which was produced as a result of this novel frameshift mutation, was predicted to be presented by this patient’s HLA-B protein. The polyclonal antibody of the synthesized 13-mer neo-peptide was produced in rabbits. Western blotting results showed a good antibody-neoantigen specificity, and Immunohistochemistry (IHC) staining with the antibody of the neo-peptide clearly differentiated neoplastic cells from normal cells. A search of the Catalogue of Somatic Mutations in Cancer (COSMIC) database also revealed that 53.2% of mutations in *BAP1* were frameshift indels with neo-peptide formation. An identified tumor-specific neo-antigen could be the potential molecular biomarker for personalized diagnosis to precisely subtype rare malignancies such as MPM.

## 1. Introduction

Malignant mesothelioma (MM) is a rare and highly aggressive tumor that is usually associated with asbestos exposure. The reported incidence of MM in the US is 1 per 100,000 [1]. Only 10% cases are peritoneal MM, and half of the patients do not have a history of asbestos exposure [2]. Patients with peritoneal MM are resistant to all available radio- and chemotherapies and die within two years of diagnosis. There is no effective clinical protocol for the treatment of this disease.

The current first-line treatment for patients with MM is platinum-containing regimens. Unfortunately, nearly half of the patients do not respond to this therapy. It is difficult to evaluate the clinical response rate to the novel therapies according to classical RECIST criteria because of MM’s histologic and molecular heterogeneity [3]. Patients with MM have not yet benefited from individualized treatment, such as targeted drugs.

The understanding of molecular pathobiology of MM has recently evolved. A whole genome analysis of malignant peritoneal mesothelioma (MPM) has been previously reported [4]. The biggest study of the genome-wide mutational landscape of peritoneal MM only collected 12 samples. Among mutations in known mesothelioma-related genes, such as *NF2* and *CDKN2A*, somatic mutations in *BAP1* are the most common recurrent events [5,6,7]. The molecular pathway and mechanism still remains unknown due to a lack of large-scale case studies. However, personalized tumor-specific neo-antigen profiling associated with immunotherapy might be a suitable strategy for MPM patients.

The use of tumor-specific neo-antigens as targets for cancer immunotherapy has become a powerful strategy for the treatment of chronic lymphocytic leukemia [8] and metastatic cholangiocarcinoma [9]. Next-generation sequencing (NGS) coupled with somatic mutations bioinformatics analysis allows for the screening of tumor-specific mutated proteins. Compared to point mutations, the novel open reading frames (neoORFs) generated by small inserts or deletions could induce highly specific antitumor immunity and could be recognized by T cells [10]. Hacohen and his team also emphasized that neoORFs should be prioritized because they provide a completely novel protein sequence with no counterpart in any normal cells [11].

**Figure 1:**
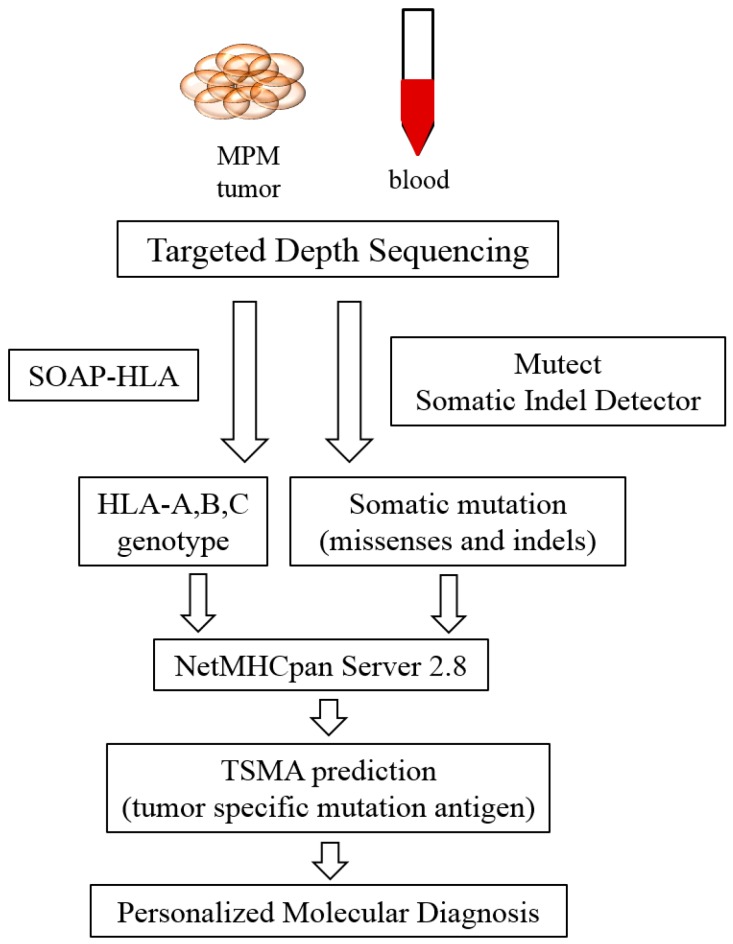
Pipeline for tumor specific neo-antigen analysis: next generation sequencing on data from tumor and blood DNA samples is filtered by Mutect and Somatic Indel Detector software. The HLA genotype is extracted from next-generation sequencing (NGS) data by SOAP-HLA. NetMHCpan server 2.8 is a common tool for the prediction of the binding ability of mutant peptides-HLA.

The pipeline of individual neo-antigen profiling in MPM is described in Figure 1: paired NGS data of tumor tissue DNA and blood genome DNA is used to filter somatic mutations. Human leukocyte antigen (HLA) genotyping is analyzed by SOAP-HLA software (Beijing Genomics Institute, Beijing, China). NetMHCpan is a major tool for the prediction of MHC binding. Once the tumor-specific neo-antigens are validated, they are candidate targets for the design of individualized therapy.

Here, we performed 725-oncogene depth targeted sequencing on a tumor sample and its paired peripheral blood DNA from a patient with malignant peritoneal mesothelioma. After bioinformatics analyses of somatic mutations and prediction of MHC presentation, we validated a novel somatic insert frameshift variation in *BAP1*. The antibody of this neo-antigen succeeded to differentiate the tumor cells from normal cells during pathological diagnosis. Compared to the reported somatic frameshift mutations of *BAP1* in the Catalogue of Somatic Mutations in Cancer (COSMIC) database, the neo-antigen of the Bap1 protein is an ideal biomarker for molecular diagnosis and precisely subtyping of MM.

## 2. Results

### 2.1. Next-Generation Sequencing (NGS) Data Analysis

The targeted genomic region is approximately 3 Mbp in size; approximately 1.6 Gbases of sequence data remained per sample after the removal of low-quality reads. The deep targeted sequencing achieved a mean depth of coverage of 533-fold. A total of 2897 somatic base substitutions and 218 somatic small indels were called somatic variants compared to blood DNA sequencing data (Table 1). First, the total variants were filtered by their position (in the coding region), the type of mutation (non-synonymous or frameshift), the number of reads (at least 3 reads of mutated alleles in tumors) and percentage of mutant reads (0% in compared DNA). We observed only 88 somatic mutations and 9 somatic indels compared to peripheral blood DNA. Second, the non-annotated variants in dbSNP and 1000Genome and their further functional prediction by Sorting Intolerant From Tolerant (SIFT) or Polymorphism phenotyping (Polyphen) showed that “damaged” segments were kept. The percentage of mutated reads was the last filtering criteria applied.

### 2.2. Somatic Mutation Filtration and Validation

All variants with a percentage of mutant reads >5% were chosen for further validation and are listed in Table 2. All the variants with >5% percentage of mutant reads were listed in Table 2. Only 5 mutations were confirmed by the Sanger method to be somatic variants of MPM in this case: mutations in *NOTCH2* (exon13, c.2108G > A, p.G703D), *PDE4DIP* (exon1, c.862C > A, p.L288M), *BAP1* (exon13, c.1568_1569insTGTC, p.V523fs), *ATP10B* (exon7, c.628G > A, p.E210K), and *NSD1* (exon23, c.7445A > C, p.K2482T).

The remaining non-validated 5 variants had a relatively low percentage of mutant reads (<10%) except for *MN1* and *PRG4*. However, the number of mutant reads in *MN1* and *PRG4* was 3/total 14 reads and 6/total 33 reads, respectively, which suggested a poor coverage depth in these two positions. Although the Sanger method has limited sensitivity due to the rare mutant allele copies in heterogeneous cancer DNA samples, *MN1* and *PRG4* remain potential candidates responsible for the development and progress of malignant mesothelioma. We also identified a second somatic mutation in filtered *NSD1* (exon 7, c.3982A > G, p.K1328E) with a low percentage of mutant reads (5.08%). This variant was not confirmed by the Sanger method; however, two mutations in the same gene highlighted the importance and involvement of this gene in our case.

### 2.3. Prediction of Bap1 Neo-Peptide’s Protein Function and Major Histocompatibility Complex (MHC) Presentation

**Figure 2:**
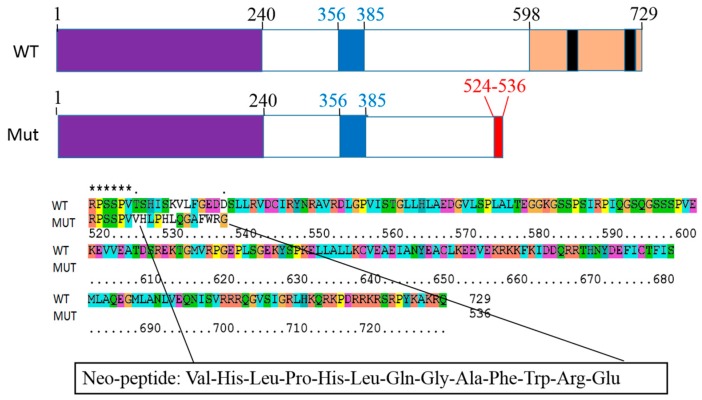
Functional domain of the Bap1 protein: domain in purple: ubiquitin C-terminal hydrolase, domain in blue: HCF-binding motif, domain in peach: nuclear localization; the sequence of neoORF (red: 524th–536th aa is the sequence of the 13-mer neo-peptide); * asterisk: same acids in both sequences.

Among the 5 confirmed mutations, 4 missense mutations in *NOTCH2*, *PDE4DIP*, *ATP10B* and *NSD1* caused only one amino acid substitution in protein sequence and did not change the length of the respective proteins. The 4 bases insertion in *BAP1* resulted in a stop codon 13-amino acids (aa) after the insertion site (523th aa). The mutated protein was 536-aa in length in comparison to 729-aa the wild-type Bap1 protein (Figure 2). The sequence of last 13-mer neoORF was VHLPHLQGAFWRG. This neo-peptide was not homologous with any human protein according to the protein Basic Local Alignment Search Tool (BLAST) result.

The HLA genotype of this patient was reported as follows: HLA-A*11:12, HLA-B*35:42 and HLA-C*04:01. The MHC class-I binding affinity across all possible 8-mer to 11-mer peptides of wild-type and mutant proteins was predicted using NetMHCpan 2.8 Server. The prediction results according to patient’s HLA genotype are shown in Table 3.

Seven of 25 peptides were predicted to have high affinity (strong binding, half maximal Inhibitory Concentration (IC_50_) <150 nM) to HLA-A or HLA-B molecules. Only 3 of 7 peptides with mutated sites were predicted to specifically bind to HLA molecules: neo 8-mer LPHLQGAF peptide of mutated Bap1 (IC_50_ = 17.46 nM), 11-mer KTNLKQRCVVK of mutated Atp10b (IC_50_ = 135.45 nM), and 8-mer TPQADETM of mutated Nsd1 (IC_50_ = 78.89 nM).

### 2.4. Western Blotting and Immunohistochemistry (IHC) Results

All three mutant neo-peptides predicted to bind to HLA molecules were synthesized. To obtain a sufficient quantity specific antibody for the western blotting and IHC study, polyclonal antibodies against neo-peptides were rapidly produced in rabbits. Only the 8-mer LPHLQGAF peptide of mutant Bap1 provoked a strong immune response in rabbits.

**Figure 3:**
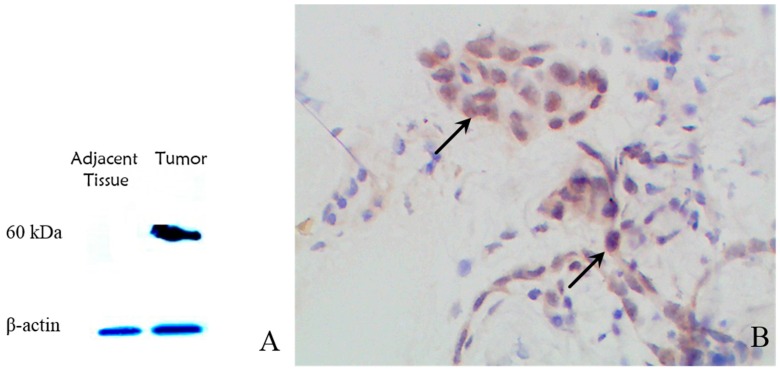
(**A**) Western blotting results showed a 60-kDa protein in tumor tissue correlating to the mutant truncated protein (β-actin as internal control); (**B**) Immunohistochemistry results showed heavy staining of neoplastic cells (black arrows); magnification: 400×.

The results of western blotting on tumor and adjacent tissue proteins using polyclonal antibody are shown in Figure 3A. An estimated 60-kDa protein correlated to the 523-aa mutant Bap1 (compared to the 80 kDa wild-type Bap1 protein) was observed. We did not find the same protein in adjacent tissue samples, which suggested a good specificity of this polyclonal antibody for mutant Bap1. IHC results demonstrated neo-peptide expression and the existence of mutant Bap1, especially because the mesothelial neoplastic cells were intensely stained (Figure 3B). However, the localization of the neo-antigen on the cell’s surface was not proven in IHC results.

## 3. Discussion

The 725-oncogene depth targeted sequencing revealed four confirmed base-substitution somatic mutations in *NOTCH2*, *NSD1*, *PDE4DIP*, and *ATP10B* and one verified 4-base insert frameshift somatic mutation in *BAP1*. According to the results of the functional prediction, the mutations in *BAP1*, *NOTCH2* and *NSD1* were the most possible driver somatic mutations in the development of MM in our patient.

The evolutionarily conserved Notch signaling pathway is necessary for the determination of cell differentiation, proliferation and survival. The NOTCH family acts as both oncogenes and tumor suppressor genes in many malignancies, such as breast cancer, cervical cancer and colorectal cancer [12]. The Notch signaling pathway could regulate gene expression via the activation of the Ras/Raf/Mapk signaling pathway, which is reported to be the most dysregulated pathway in cancer [13]. A prolonged Mapk1/2 activation after exposure to asbestos in primary murine pleural cell culture has been shown [14].

Nsd1 is Su(var)3-9, Enhancer-of-zeste and Trithorax(SET) domain-containing histone methyltransferase that plays a key role in the regulation of transcription by histone modification. The germline abrogation in *NSD1* results in a childhood overgrowth syndrome called Sotos syndrome (OMIM 117550) [15]. The somatic mutations in *NSD1* have been identified in multiple myelomas, lung cancer, neuroblastomas and glioblastomas [16]. The pathways of Nsd are not well understood; however, it is becoming a novel anti-tumor target because of its role in tumorigenesis.

Considering the results of several studies that identified recurrent genetic alternations in MMs that could be “driver” mutations, this frameshift mutation in *BAP1* seems to be the best candidate [17,18,19,20]. *BAP1* encodes a ubiquitin carboxy-terminal hydrolase whose substrate is a small regulatory protein involved in apoptosis, the cell cycle, cell division, DNA transcription, and DNA repair. The DNA damage induced by asbestos fibers should be repaired in mesothelial cells. One of four DNA repair systems is nucleotide excision repair (NER), which requires strict regulation. Ubiquitylation seems to be a key regulator of NER [21]. The C-terminal glycine residue of ubiquitin is covalently attached to an ε-amino group of lysine residues in substrate proteins. The linkage between ubiquitin and substrate impacts different cell processes such as proteasomal degradation, cellular signaling and protein-protein interaction [22,23].

**Figure 4:**
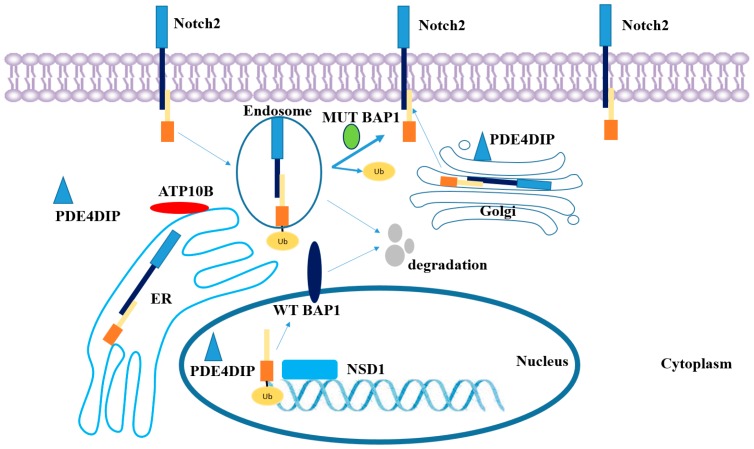
Notch signaling pathway and the localization of NSD1, BAP1, PDE4DIP and ATP10B in mesothelial cells.

In this case, the mutant Bap1 protein lost its nuclear localization function, which caused aberrant enzymatic activity in cytoplasm. The theoretical hypothesis is presented in Figure 4: Mutant Bap1 protein continues to deubiquitylate ubiquitous Notch signaling complexes, which are supposed to be degraded after ubiquitylation. Meanwhile, the decreased ubiquitylation of transcription factors, such as Nsd1*,* dysregulates histone modification. The excessive activation of the Notch signaling pathway and the resulting dysfunction in transcription could initiate primary carcinogenesis. Further expression-level studies of *NOTCH* and *NSD1* should facilitate the understanding of the pathological mechanism of MPM.

**Figure 5:**
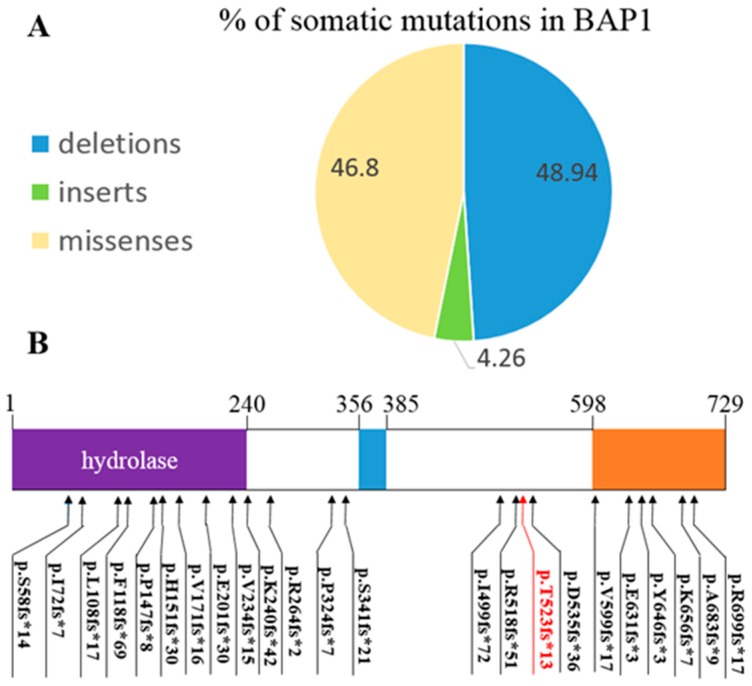
(**A**) Percentages of somatic mutations in *BAP1* gene: missense point mutations, 46.8%; deletions, 48.94%; and inserts, 4.26%; (**B**) Mutations’ position along the Bap1 protein after frameshift (22 frameshift mutations of COSMIC in black, novel frameshift in red).

A small number of clinical cases of MPM have been discovered and reported; we searched for these cases in COSMIC. Only 10 peritoneal tissue samples were included in the database of COSMIC v. 76, with no mutational information about *BAP1*, while the mutation rate in pleural tissue samples was up to 27.22% (89 samples/327 total tested). Only 47 mutations are amino acid-changing: 46.8% missense and 53.2% small inserts and deletions (Figure 5A). Twenty-two indels of 25 can induce a stop codon after frameshift site and terminate protein translation. Figure 5B presented the positions of all frameshift mutations along the protein. Compared to all variants in *BAP1* regardless of tumor type, 134/532 (25.2%) variants are frameshift mutations. All frameshift mutations directly influenced two Bap1 functional domains: the hydrolase domain as the enzymatic activity core and nuclear localization domain as the nuclear membrane anchor. Their mutational positions suggested two different types of Bap1 aberrances: total loss of Bap1 protein or excessive enzymatic activity in the cytoplasm without transportation to the nuclear membrane.

In addition to targeting signaling pathway changes in cancer, tumor-specific neo-antigen-based immunotherapy combined with the checkpoint inhibitor PD-1 have been developed [24,25,26]. The critical step to T cell activation is the recognition of the cancer neo-antigen and MHC-I complex [27]. Algorithms such as NetMHCpan are common tools for affinity prediction. However, the validation of a potential neoantigen as true target is still limited by the lack of a suitable cellular model with the correlated HLA genotype.

The mutations in *BAP1* were the most common events not only in MM patients but also in other types of cancer, such as uveal melanoma [28] and renal cancer [29]. The discovery of a universal biomarker or a therapeutic target is difficult because of the tumors’ heterogeneity, while personalized genome analysis and neo-antigen profiling allow for the development of novel diagnostic biomarkers and clinical therapies. The germline mutation in *BAP1* was recommended for screening for hereditary case diagnosis [30]. For sporadic MM patients with no obvious clinical treatment, the tumor-specific neo-antigen caused by frameshift mutations of Bap1 could be identified based on patient’s sequencing results and the prediction of its MHC-binding ability. The Bap1 protein’s neo-antigen has potential for the personalized molecular diagnosis and precise subtyping of malignancies such as mesothelioma.

## 4. Materials and Methods

### 4.1. Case Sample

The patient was a 68-year-old female with a history of asbestos exposure who presented with a chief complaint of nausea and abdominal pain. Cross-sectional imaging results identified peritoneal lesions. Three cycles of systemic chemotherapy with cisplatin and pemetrexed were initiated after surgical resection. The patient underwent cytoreductive surgery with hyperthermic intraperitoneal chemotherapy. She was diagnosed with an epithelial type peritoneal malignant mesothelioma characterized by the following clinical features: CK5/6 (+++), CK7 (+++), calretinin (++), D2-40 (+), Vim (++), CK (++), ER (−), PR (−), CEA (−), Ki67 (<10% +). The primary malignant peritoneal mesothelioma tumor sample and adjacent tissue from surgical resection were collected and stored in liquid nitrogen. The matched peripheral blood sample was also collected. Written consent was obtained for the collection and use of tissues for research purposes, with ethical approval from Research Ethics Committee of Zhejiang University School of Medicine. 

### 4.2. Deep Targeted Sequencing

The DNA of the tissue was extracted from fresh-frozen samples and blood. Whole genomic DNA libraries were prepared to generate an average insert size of 350 bp according to the manufacturer’s protocols (Illumina, CA, USA). Captured and enriched targeted libraries using the commercial GenCapTM oncocap_725 kit (MyGenostics, Beijing, China) were sequenced with the Hiseq 2500 platform. The GenCapTM oncocap_725 kit targeted 725 tumor-related genes (see Supplemental Material Table S1). These genes are the most frequently reported genes involved in carcinogenesis and tumor development.

### 4.3. NGS Data Processing and Prediction of MHC Binding

The raw data were cleaned and filtered by their Quality Control (QC), and the reads were aligned to the reference genome version hg19 using Burrows-Wheeler Aligner (BWA) software. To remove the redundant duplications produced by PCR, Sequence Alignment/Map tools (SAMtools) were used, followed by the detection of Single Nucleotide Polymorphisms (SNP) and short insertions or deletions using the Genome Analysis Toolkit (GATK). The software ANNOVAR was used to complete annotation and classification. All variants were overlapped to the dbSNP138 and 1000Genomes database to mask the common or germline polymorphisms. An algorithm MuTect was used to identify somatic single nucleotide variants (SNVs) and indels (insert and deletion) in sequencing data. Then, the results were compared to the COSMIC database. SIFT, Polyphen, MutationTaster and GERP++ were used to predict the functions of the non-silent mutations. The percentage of mutant reads was calculated by alternative read number/(reference read number + alternative read number) × 100%.

The HLA-A, -B and -C loci genotypes were obtained from local hospital according to the HLA SSP genotyping results. The MHC class I binding affinity was predicted across possible 8- and 11-mer peptides of mutated tumor peptides by NetMHCpan 2.8 Server (www.cbs.dtu.dk/services/NetMHCpan/). An IC_50_ (nM) < 150 nM was considered to represent strong binding (high affinity).

### 4.4. Somatic Mutation Validation by PCR

The somatic SNVs and indels chosen by data processing were verified at the transcriptional level. The total RNA was extracted and cDNA was synthesized according to the manufacturer’s protocols (Qiagen, Frankfurt, Germany). The specific primers were designed by Oligo 6.0 (see Supplemental Material Table S2). Reverse transcription-PCR amplification was carried out using 1.5 mM MgCl_2_, 1.25 U Taq DNA polymerase (Takara, Japan), 0.25 μM primer and 50 ng cDNA. After the initial cycle of denaturation at 94 °C for 5 min, 30 cycles were continued, which consisted of denaturation at 94 °C for 30 s, annealing at 60 °C (±3 °C) for 1 min, extension at 72 °C for 1 min and final extension for 7 min at 72 °C on an Eppendorf Mastercycler Pro Thermal Cycler. The PCR products were sent to Sangon Biotech Company (Shanghai, China) to sequencing. The obtained amplicon sequences were compared to the reference sequence to validate the mutations.

### 4.5. Neo-Peptide Synthesis and Polyclonal Antibody Production

The neo-peptide was synthesized by the Chinese Peptide Company (Hangzhou, Zhejiang, China) with a purity >95%. Polyclonal antibodies for the neo-peptide were produced with the following protocol: briefly, New Zealand rabbits were immunized by subcutaneous injection of a solution containing 100 μg peptide with Freund’s complete adjuvant for the primary dose and 50 μg peptide with Freund’s incomplete adjuvant for 3 booster doses. The quality of the antibodies in serum (humoral immune response) was monitored by indirect enzyme linked immunosorbent assay (ELISA). Then, the rabbit serum was collected and polyclonal antibody was purified by Protein A affinity chromatography (General Electric Company, Fairfield, CA, USA).

### 4.6. Western Blot Study and Immunohistochemistry (IHC) 

The total protein extracts of tissues (tumor and adjacent tissue) were separated using sodium dodecyl sulfate, sodium salt-polyacrylamide gel electrophoresis (SDS-PADE), transferred to nitrocellulose membranes and stained with neo-peptide-specific antibody. The immunohistochemistry of the mesothelioma tumor followed the protocol as previously described [8]: the paraffin blocks with mesothelioma tissues were cut into 6-μm sections and mounted onto positively charged microscope slides, and 3% H_2_O_2_ in PBS was used for 20 min to block endogenous peroxidase activity. Primary antibody of Bap1 neo-antigen (1:2000 dilution) was added, followed by the biotinylated secondary antibodies (Beyotime, Nantong, Jiangsu, China) for 40 min. Finally, sections were stained with diaminobenzidine (DAB) and counterstained with hematoxylin. Research manuscripts reporting large datasets deposited in a publicly available database should specify where the data have been deposited and provide the relevant accession numbers. If the accession numbers have not yet been obtained at the time of submission, please state that they will be provided during review. They must be provided prior to publication.

## 5. Conclusions

We reported a novel 4-base insert somatic mutation in *BAP1* in a MM patient’s tumor sequencing results. This frameshift mutation was translated into a truncated protein with a 13-mer neo-peptide tail, which was predicted to be presented by the patient’s HLA-B molecule as a tumor-specific neo-antigen. The neo-antigen provoked a strong immune response in rabbits. The polyclonal antibody succeeded to stain the neoplasm on IHC. Frameshift mutations in *BAP1* are common events in MM, and the neo-antigens could be ideal biomarkers for diagnosis.

## Figures and Tables

**Table 1:** Number of somatic variants after applying different selection criteria.

Type of Mutation	Compared to Peripheral Blood
	Total Variants
Base Substitution	2897
Small Indel	218
	In the coding region, variant reads >3, 0% mutation frequency in blood
Base Substitution	88
Small Indel	9
	Mutation frequency >1% in tumor, non-repertoire in dbSNP and 1000Genome
Base Substitution	24
Small Indel	4
	Mutation frequency >5%, “causing disease” in SIFT and PolyPhen prediction
Base Substitution	8
Small Indel	2
	Validation by RT-PCR
Base Substitution	4
Small Indel	1

**Table 2:** List of somatic mutations (percentage of mutant reads >5%).

Chromosome	Begin Position	End Position	Associated Gene	Reference	Alternative	Tumor Frequency	Mutation_Type	Validation by Sanger Sequencing
1	120497774	120497774	*NOTCH2*	C	T	17.63%	nonsynonymous	Yes
1	144930847	144930847	*PDE4DIP*	G	T	8.82%	nonsynonymous	Yes
1	186276044	186276046	*PRG4*	CCA	–	17.65%	nonframeshift	No
3	12626480	12626480	*RAF1*	T	C	8.11%	nonsynonymous	No
3	52437592	52437592	*BAP1*	–	GACA	21.21%	frameshift	Yes
5	160097517	160097517	*ATP10B*	C	T	12.28%	nonsynonymous	Yes
5	176665298	176665298	*NSD1*	A	G	5.08%	nonsynonymous	No
5	176721814	176721814	*NSD1*	A	C	15.89%	nonsynonymous	Yes
14	95599707	95599707	*DICER1*	A	G	5.77%	nonsynonymous	No
22	28193936	28193936	*MN1*	C	A	15.38%	nonsynonymous	No

**Table 3:** All possible peptides bound to HLA-A, B molecules were listed: amino acids in green are mutant sites. Peptide sequences in red were predicted to have a strong binding ability to HLA molecules and are potential tumor-specific neo-antigens.

HLA Typing	Peptide Sequence	Peptide Type	1-log50k	nM	Rank	Banding Type
HLA-A*11:12	ATCINGVNGFR	NOTCH2-703WT	0.525	171.19	1.50	Weak Binding
HLA-A*11:12	CINGVNGFR	NOTCH2-703WT	0.482	271.44	2.00	Weak Binding
HLA-A*11:12	ATCINGVNDFR	NOTCH2-G703D	0.5	224.39	2.00	Weak Binding
HLA-A*11:12	CINGVNDFR	NOTCH2-G703D	0.479	280.08	2.00	Weak Binding
HLA-A*11:12	KLELALSMIK	PDE4DIP_288WT	0.476	290.35	2.00	Weak Binding
HLA-A*11:12	ELALSMIK	PDE4DIP_288WT	0.446	402.87	3.00	Weak Binding
HLA-A*11:12	KMELALSMIK	PDE4DIP_L288M	0.528	165.47	1.50	Weak Binding
HLA-A*11:12	ELALSMIK	PDE4DIP_L288M	0.446	402.87	3.00	Weak Binding
HLA-B*35:42	HKMELALSM	PDE4DIP_L288M	0.525	171.29	2.00	Weak Binding
HLA-A*11:12	ASLDGETNLK	ATP10B_210WT	0.642	47.93	0.80	Strong Binding
HLA-A*11:12	SLDGETNLK	ATP10B_210WT	0.523	174.12	1.50	Weak Binding
HLA-A*11:12	TASLDGETNLK	ATP10B_210WT	0.451	380.57	3.00	Weak Binding
HLA-A*11:12	ASLDGKTNLK	ATP10B_E210K	0.638	50.5	0.80	Strong Binding
HLA-A*11:12	KTNLKQRCVVK	ATP10B_E210K	0.546	135.45	1.50	Strong Binding
HLA-A*11:12	SLDGKTNLK	ATP10B_E210K	0.515	190.94	2.00	Weak Binding
HLA-A*11:12	TASLDGKTNLK	ATP10B_E210K	0.443	413.1	3.00	Weak Binding
HLA-B*35:42	MPVLESSSW	NSD1_2482WT	0.755	14.22	0.30	Strong Binding
HLA-B*35:42	MPVLESSS	NSD1_2482WT	0.497	232.2	2.00	Weak Binding
HLA-B*35:42	MPVLESSSW	NSD1_K2482T	0.755	14.22	0.30	Strong Binding
HLA-B*35:42	TPQADETM	NSD1_K2482T	0.596	78.89	1.00	Strong Binding
HLA-B*35:42	MPVLESSS	NSD1_K2482T	0.497	232.2	2.00	Weak Binding
HLA-B*35:42	TPQADETMPVL	NSD1_K2482T	0.451	379.75	3.00	Weak Binding
HLA-B*35:42	LPHLQGAF	BAP1_V523fs	0.736	17.46	0.40	Strong Binding
HLA-B*35:42	LPHLQGAFW	BAP1_V523fs	0.492	242.83	2.00	Weak Binding
HLA-B*35:42	SPVVHLPH	BAP1_V523fs	0.465	326.57	3.00	Weak Binding

## Supplementary Materials

Supplementary materials can be found at http://www.mdpi.com/1422-0067/17/5/739/s1.

## Abbreviations

MPM	Malignant peritoneal mesothelioma
NGS	Next generation sequencing
MHC	Major histocompatibility complex
HLA	Human leukocyte antigen
