# Supplementary material for: A Tumor-Specific Neo-Antigen Caused by a Frameshift Mutation in BAP1 Is a Potential Personalized Biomarker in Malignant Peritoneal Mesothelioma
Source: Int J Mol Sci. 2016 May 14;17(5):739. doi: 10.3390/ijms17050739 (PMC4881561; doi:10.3390/ijms17050739)
Supplement: Supplementary file 1 [file ijms-17-00739-s001.pdf]

Supplemental Material: A Tumor-Specific Neo-Antigen Caused by a Frameshift Mutation in *BAP1* is a Potential Personalized Biomarker in Malignant Peritoneal Mesothelioma

Jun Lai, Zhan Zhou, Xiao-Jing Tang, Zhi-Bin Gao, Jie Zhou and Shu-Qing Chen

Table S1. 725 targeted genes in GenCap™ oncocap_725 kit.

ABI1	*ABI3*	*ABL1*	*ABL2*	*ABLIM1*	*ACBD6*	*ACCS*	*ACSL3*	*ACSL6*	*ACVR1B*
ADAM12	*ADAM19*	*ADAM29*	*ADAMTS16*	*ADAMTS18*	*ADAMTS20*	*ADAMTSL3*	*AFF1*	*AFF3*	*AIM1*
AKAP12	*AKAP9*	*AKT1*	*AKT2*	*AKT3*	*ALK*	*APC*	*APC2*	*APOL1*	*ARF1*
ARHGAP26	*ARHGEF12*	*ARID1A*	*ARNT*	*ARRB2*	*ASF1A*	*ASPSCR1*	*ASXL1*	*ATF1*	*ATIC*
ATM	*ATP10B*	*ATP13A3*	*BAG2*	*BAP1*	*BCL10*	*BCL11A*	*BCL11B*	*BCL2*	*BCL2L1*
BCL3	*BCL6*	*BCL7A*	*BCL9*	*BCR*	*BHLHE40*	*BIN1*	*BIRC3*	*BLM*	*BMI1*
BMPR1A	*BNIP3L*	*BRAF*	*BRCA1*	*BRCA2*	*BRD3*	*BRD4*	*BRD7*	*BRIP1*	*BTG1*
BUB1	*BUB1B*	*C15orf55*	*C18orf32*	*C2orf40*	*CANT1*	*CAPN1*	*CARD10*	*CARD11*	*CARD8*
CARS	*CASC5*	*CASP1*	*CASP8*	*CBFA2T3*	*CBFB*	*CBL*	*CBLB*	*CBLC*	*CBX8*
CCDC6	*CCL2*	*CCL26*	*CCNA2*	*CCNB1*	*CCNB1IP1*	*CCND1*	*CCND2*	*CCND3*	*CCNDBP1*
CD27	*CD3EAP*	*CD74*	*CDC25C*	*CDC42EP1*	*CDC6*	*CDC73*	*CDH1*	*CDH10*	*CDH11*
CDK1	*CDK2*	*CDK4*	*CDK5*	*CDK6*	*CDKN1A*	*CDKN1B*	*CDKN1C*	*CDKN2A*	*CDKN2AIP*
CDKN2B	*CDKN2C*	*CDKN2D*	*CDX2*	*CEBPA*	*CEP110*	*CHCHD7*	*CHEK1*	*CHEK2*	*CHIC2*
CHN1	*CIC*	*CIITA*	*CIRBP*	*CITED2*	*CLDN1*	*CLP1*	*CLTC*	*CLTCL1*	*CNBP*
COL1A1	*COMMD2*	*COMMD3*	*COMMD4*	*COMMD5*	*COMMD6*	*COMMD8*	*COX6C*	*CRAF*	*CREB1*
CREB3L2	*CREBBP*	*CREG1*	*CRTC1*	*CRTC3*	*CSF1R*	*CTNNA1*	*CTNNA2*	*CTNNB1*	*CTSF*
CTSW	*CXCL1*	*CXCR7*	*CYB561*	*CYLD*	*DAP3*	*DARC*	*DCLRE1B*	*DDB2*	*DDIT3*
DDX10	*DDX5*	*DDX6*	*DEK*	*DICER1*	*DLEC1*	*DMTF1*	*DNMT3A*	*DUSP3*	*DUSP4*
DUSP6	*DYRK1A*	*E2F1*	*E2F3*	*EDF1*	*EFEMP1*	*EGFR*	*EIF4A2*	*ELF4*	*ELK4*
ELL	*ELN*	*EML4*	*ENDOG*	*EP300*	*EPHA3*	*EPHA4*	*EPHA7*	*EPHB6*	*EPS15*

ERBB2	*ERC1*	*ERCC2*	*ERCC3*	*ERCC4*	*ERCC5*	*ERG*	*ETS1*	*ETS2*	*ETV4*
ETV5	*EWSR1*	*EXT1*	*EXT2*	*EZH2*	*EZR*	*FAM123B*	*FANCA*	*FANCC*	*FANCD2*
FANCE	*FANCF*	*FANCG*	*FAS*	*FBX031*	*FBXW7*	*FCGR2B*	*FCGR3A*	*FCHSD1*	*FCRL4*
FEV	*FGFR1OP*	*FGFR2*	*FGFR3*	*FH*	*FHL2*	*FIP1L1*	*FLCN*	*FLI1*	*FLT3*
FLT4	*FN1*	*FOXA1*	*FOXL2*	*FOXO1*	*FOXO3*	*FOXO4*	*FOXP1*	*FRA6F*	*FSTL3*
FUS	*GADD45A*	*GAS7*	*GATA1*	*GATA2*	*GLI1*	*GLI3*	*GMPS*	*GNAQ*	*GNAS*
GNG11	*GOLGA5*	*GOPC*	*GPC3*	*GPHN*	*GRIN2A*	*GUCY1A2*	*HEPACAM*	*HERPUD1*	*HIP1*
HIRA	*HIST1H4I*	*HLF*	*HMGA1*	*HMGA2*	*HMOX1*	*HMOX2*	*HNF1A*	*HNRNPA2B1*	*HOOK3*
HOXA11	*HOXA13*	*HOXA9*	*HOXC11*	*HOXC13*	*HOXD11*	*HOXD13*	*HPV6AI1*	*HRAS*	*HSP90AA1*
HSP90AB1	*HSPA9*	*ID1*	*ID2*	*IDH1*	*IDH2*	*IER3*	*IFI16*	*IGFBP3*	*IGFBP7*
IKBKB	*IKBKE*	*IKBKG*	*IL12A*	*IL1R1*	*IL2*	*IL20*	*IL21R*	*IL6ST*	*INF2*
INK4A	*INTS4*	*IRAK1*	*IRAK1*	*IRAK2*	*IRF1*	*IRF2*	*IRF3*	*IRF4*	*IRF5*
IRF7	*IRF8*	*IRS4*	*ISG15*	*ITCH*	*ITGAD*	*ITGAE*	*ITGB2*	*ITGB3BP*	*ITK*
JAK2	*JAK3*	*JAZF1*	*JUNB*	*JUND*	*KDM5C*	*KDM6A*	*KDR*	*KDSR*	*KIAA1549*
KIT	*KL*	*KLF6*	*KLK2*	*KLRG1*	*KRAS*	*KSR1*	*KSR2*	*KTN1*	*LASP1*
LCK	*LCP1*	*LDB3*	*LEO1*	*LHFP*	*LIFR*	*LIMS1*	*LIMS2*	*LIMS3*	*LIMS3-LOC440895*
LIMS3L	*LMNA*	*LMO1*	*LMO2*	*LOC651644*	*LOXL1*	*LOXL2*	*LOXL3*	*LOXL4*	*LPP*
LYL1	*LYL1*	*MAD2L1BP*	*MAF*	*MAFB*	*MALT1*	*MAML2*	*MAP2K3*	*MAP2K4*	*MAP2K7*
MAP3K14	*MAP3K3*	*MAP3K8*	*MAPK14*	*MAPKAPK5*	*MAPKBP1*	*MCM2*	*MDM4*	*MECOM*	*MEN1*
MET	*MIR146A*	*MIR17*	*MIR205*	*MIR217*	*MIR22*	*MIR29A*	*MIR30A*	*MIR34A*	*MIR34B*
MIR34C	*MIR93*	*MITF*	*MKL1*	*MLF1*	*MLH1*	*MLL*	*MLL2*	*MLL3*	*MLLT1*
MLLT10	*MLLT11*	*MLLT3*	*MLLT4*	*MLLT6*	*MMP2*	*MN1*	*MNX1*	*MORF4*	*MPL*
MSH2	*MSH6*	*MSI2*	*MSN*	*MT-CYB*	*MTCP1*	*MTOR*	*MUC1*	*MUTYH*	*MXD3*
MXD4	*MYC*	*MYCL1*	*MYCN*	*MYD88*	*MYH11*	*MYH9*	*MYST3*	*MYST4*	*NACA*
NBN	*NCOA1*	*NCOA2*	*NCOA4*	*NDFIP1*	*NF1*	*NF2*	*NFKB1*	*NFKB2*	*NFKBIA*
NFKBIB	*NFKBID*	*NFKBIE*	*NFKBIL1*	*NFKBIZ*	*NFRKB*	*NIN*	*NINJ1*	*NKAP*	*NKAPL*
NKAPP1	*NKIRAS1*	*NKIRAS2*	*NLRP2*	*NOD1*	*NOD2*	*NONO*	*NOTCH1*	*NOTCH2*	*NOX4*
NPM1	*NR4A3*	*NRAS*	*NSD1*	*NTRK1*	*NTRK3*	*NUAK1*	*NUMA1*	*NUP214*	*NUP98*

NUTF2	*OLIG2*	*OMD*	*PAFAH1B2*	*PALB2*	*PATZ1*	*PAX3*	*PAX5*	*PAX7*	*PAX8*
PBRM1	*PBX1*	*PCSK7*	*PDCD11*	*PDCD4*	*PDE4DIP*	*PDGFB*	*PDGFRA*	*PDGFRB*	*PEA15*
PER1	*PHOX2B*	*PIAS4*	*PICALM*	*PIK3CA*	*PIK3CG*	*PIK3R1*	*PIM1*	*PINK1*	*PLA2R1*
PLAG1	*PLEKHG5*	*PML*	*PMS1*	*PMS2*	*POU2AF1*	*POU5F1*	*PPARG*	*PPM1A*	*PPP1R13L*
PPP1R3A	*PPP2R1A*	*PPP2R2C*	*PPP4C*	*PRCC*	*PRDM16*	*PRF1*	*PRG4*	*PRKAR1A*	*PRKCZ*
PRKD1	*PRPF19*	*PSD*	*PTCH1*	*PTEN*	*PTK2*	*PTPN11*	*PTPN22*	*PTPRU*	*PTTG1*
RABEP1	*RAD51L1*	*RAF1*	*RANBP17*	*RANBP2*	*RAP1A*	*RAP1GAP*	*RAP1GDS1*	*RARA*	*RARB*
RASEF	*RASGRF2*	*RB1*	*RBBP8*	*RBL1*	*RBL2*	*RBM15*	*RBX1*	*REL*	*RELA*
RELB	*RET*	*RGN*	*RHOH*	*RIPK1*	*RNF25*	*RNF43*	*ROMO1*	*ROS1*	*RPL10L*
RPL22	*RPLP1*	*RPN1*	*RRAD*	*RSL1D1*	*RUNX1*	*RUNX1T1*	*S1PR2*	*SAP30*	*SBDS*
SDHB	*SDHC*	*SDHD*	*SEC31A*	*SEN2*	*SEN3*	*SEN6*	*SEN6A*	*SEN6B*	*SENP1*
5-Sep	*6-Sep*	*9-Sep*	*SET*	*SFPQ*	*SFRS3*	*SH3GL1*	*SHC1*	*SILV*	*SIRT1*
SIRT6	*SKIL*	*SLC22A9*	*SLC45A3*	*SLC50A1*	*SMAD1*	*SMAD2*	*SMAD3*	*SMAD4*	*SMAD9*
SMARCA4	*SMARCB1*	*SMO*	*SOCS1*	*SRC*	*SRGAP3*	*SS18*	*SS18L1*	*SSX1*	*SSX2*
SSX4	*STIL*	*STK11*	*SUFU*	*SUMO1*	*SUMO2*	*SUMO3*	*SUMO4*	*SUPT4H1*	*SYK*
TAB2	*TAB3*	*TAF15*	*TAGLN*	*TAL1*	*TAL2*	*TANK*	*TBK1*	*TBKBP1*	*TCEA1*
TCF12	*TCF3*	*TCF7L1*	*TCF7L2*	*TCL1A*	*TCL6*	*TEC*	*TEK*	*TERC*	*TERT*
TET1	*TFE3*	*TFEB*	*TFG*	*TFPT*	*TFRC*	*TGFB1I1*	*TGFBR2*	*TGM3*	*THRAP3*
TICAM1	*TICAM2*	*TIFAB*	*TIMM50*	*TLX1*	*TLX3*	*TMED4*	*TMEM158*	*TMPRSS2*	*TNFAIP3*
TNFRSF10B	*TNFRSF17*	*TNFSF13*	*TNFSF14*	*TNIP1*	*TNIP2*	*TOP1*	*TP53*	*TP53BP1*	*TPM3*
TPM4	*TPR*	*TRADD*	*TRAF1*	*TRAF3*	*TRAF3IP2*	*TRAF3IP2-AS2*	*TRIM24*	*TRIM27*	*TRIM33*
TRIP11	*TSC1*	*TSC2*	*TSC22D3*	*TSHR*	*TTL*	*TUBB4*	*TWIST1*	*UBN1*	*UNC5CL*
UQCRC2	*USP6*	*USP9X*	*USP9Y*	*VHL*	*WAS*	*WHSC1*	*WHSC1L1*	*WLS*	*WRN*
WT1	*XPA*	*XPC*	*YWHAB*	*YWHAE*	*YWHAG*	*YWHAH*	*YWHAQ*	*YWHAZ*	*ZBTB16*
ZBTB7A	*ZNF331*	*ZNF384*	*ZNF521*	*ZSCAN4*	–	–	–	–	–

Table S2. Primer sequences for Sanger validation.

Gene	Chromosome	Position	Primer (5′ to 3′)	Tm (°C)	Product Size (bp)
NOTCH2	1	120497774	F: TCAATAAGAAACTATCACATGGGGCAG	60.5	231
			R: CAGGGCAGAGATGTAACATTGACATT	60.4	
PDE4DIP	1	144930847	F: ACCGGAACTAACTCCATCTGTCAT	60.3	224
			R: CTGAGAGAAGTGCAAAGGAACTTGG	62.0	
MN1	22	28193936	F: CTTTGGAGCCGCTGCTACTG	61.9	222
			R: CCACCGCTTGCCAGAACAT	59.7	
BAP1	3	52437592	F: GCAGGCCTGTGCTGATGAC	61.9	219
			R: GCAATGAGAGTACAGACACGGC	61.9	
ATP10B	5	160097517	F: AGGAGGCATCTACTACTTTCCCC	62.0	220
			R: CCTCCTTTTTTCCTCTGACCCC	61.9	
NSD1	5	176721814	F: TACTATCAGCTGTGGTCCAGAC	60.1	245
			R: CTCAACAGCTCTCGGCATATGC	61.9	
RAF1	3	12626480	F: ACACAGTCAGCTACCAGCCTC	61.9	224
			R: GTGCGAAAGGACCCAACTCG	61.9	
NSD1	5	176665298	F: TACAACAATTTTGGCCTGTGGACT	58.6	223
			R: GCATGTCCACCTCCAAGTTCTG	61.9	
DICER1	14	95599707	F: GCTCCAGTATTAGTGTTCGCATTAGTA	60.5	221
			R: GTAGTAAGCTGTGCTAGAACAAAAATGC	60.5	
NF2	22	30054224	F: CCCATCTCACTTAGCTCCAATGACA	61.9	225
			R: AAGAGTCTATCGCCTTGGAATGAAGA	60.4	
BIVM-ERCC5	13	103524612	F: TCTCATTGCTGTGTAAGTAATTGTTTCC	60.5	240
			R: CTCTAATTTTGTCGAGATCAGGTTTCCC	61.9	

Table S2. *Cont.*

Gene	Chromosome	Position	Primer (5′ to 3′)	Tm (°C)	Product Size (bp)
RBBP8	18	20572853	F: CTCCTCCTCAAGAAGAATTACCTACTCG	62.2	232
			R: GACCCAAGACTGTGATGTGTGAAAAG	62.4	
PRG4	1	186276044	F: GACACGTCTACCACCCAACA	59.6	2689
			R: TGCCTTGATTGGGTACTCTCG	59.8	
